# Short stature and primary ovarian insufficiency possibly due to chromosomal position effect in a balanced X;1 translocation

**DOI:** 10.1186/s13039-015-0154-3

**Published:** 2015-07-15

**Authors:** Rita Genesio, Angela Mormile, Maria Rosaria Licenziati, Daniele De Brasi, Graziella Leone, Sara Balzano, Antonella Izzo, Ferdinando Bonfiglio, Anna Conti, Gennaro Fioretti, Selvaggia Lenta, Maria Rita Poggiano, Paolo Siani, Lucio Nitsch

**Affiliations:** Department of Molecular Medicine and Medical Biotechnology, University of Naples “Federico II”, Naples, Italy; Unit of Auxology and Endocrinology, Department of Pediatrics, AORN Santobono-Pausilipon, Naples, Italy; Medical Genetics and Pediatric Unit, Department of Pediatrics, AORN Santobono-Pausilipon, Naples, Italy; Department of Medical Genetics, University of Lausanne, Lausanne, Switzerland; Medical Genetics Unit, AORN “A. Cardarelli”, Naples, Italy

**Keywords:** *DIAPH2*, *FMR1*, Primary ovarian insufficiency, Chromosomal position effect, X chromosome translocation, Turner syndrome, X;autosome translocation

## Abstract

**Background:**

Primary ovarian insufficiency (POI) is defined as a primary ovarian defect characterized by absent menarche (primary amenorrhea), a decrease in the initial primordial follicle number, high follicle-stimulating hormone (FSH) levels and hypoestrogenism. Although the etiology of a majority of POI cases is not yet identified, several data suggest that POI has a strong genetic component. Conventional cytogenetic and molecular analyses have identified regions of the X chromosome that are associated with ovarian function, as well as POI candidate genes, such as *FMR1* and *DIAPH2*.

Here we describe a 10.5-year-old girl presenting with high FSH and luteinizing hormone (LH) levels, pathologic GH stimulation arginine and clonidine tests, short stature, pterygium, ovarian dysgenesis, hirsutism and POI.

**Results:**

Cytogenetic analysis demonstrated a balanced reciprocal translocation between the q arms of chromosomes X and 1, with breakpoints falling in Xq21 and 1q41 bands. Molecular studies did not unravel any chromosome microdeletion/microduplication, and no *XIST*-mediated inactivation was found on the derivative chromosome 1. Interestingly, through immunofluorescence assays, we found that part of the Xq21q22 trait, translocated to chromosome 1q41, was late replicating and therefore possibly inactivated in 30 % metaphases both in lymphocytes and skin fibroblasts, in addition to a skewed 100 % inactivation of the normal X chromosome. These findings suggest that a dysregulation of gene expression might occur in this region. Two genes mapping to the Xq translocated region, namely *DIAPH2* and *FMR1*, were found overexpressed if compared with controls.

**Conclusions:**

We report a case in which gonadal dysgenesis and POI are associated with over-expression of *DIAPH2* gene and of *FMR1* gene in wild type form. We hypothesize that this over-expression is possibly due to a phenomenon known as “chromosomal position effect”, which accounts for gene expression variations depending on their localization within the nucleus. For the same effect a double mosaic inactivation of genes mapping to the Xq21-q22 region, demonstrated by immunofluorescence assays, may be the cause of a functional Xq partial monosomy leading to most Turner traits of the proband’s phenotype.

## Background

Primary ovarian insufficiency (POI) is a condition caused by the absence, non functionality or early depletion of the ovarian reserve. The term is now commonly used to indicate conditions as hypergonadotropic hypogonadism, premature ovarian failure (POF) and ovarian dysgenesis [1]. POI may include either severe forms, presenting with absent pubertal development and primary amenorrhea, or milder forms with post-pubertal onset characterized by disappearance of menstrual cycles (secondary amenorrhea) and defective folliculogenesis. POI is generally characterized by low levels of gonadal hormones (estrogens and inhibins) and high levels of gonadotropins (LH and FSH) (hypergonadotropic amenorrhea) [2]. Another indicator is the anti-Mullerian hormone (AMH), which can help to assess the state of follicular senescence. It is considered a possible predictor of the ovarian reserve even before menarche. [3].

Several data indicate that POI has a strong genetic component. It is often associated with X chromosomal abnormalities [4], including X monosomy (Turner syndrome) or trisomy even in mosaic form, X chromosome deletions, inversions and balanced and unbalanced X;autosomal (X;A) translocations [5–7].

Cytogenetic and molecular analysis of POI women carrying X-rearrangements allowed the identification of a “critical region” for ovarian development and function on the long arm of the X chromosome, ranging from Xq13.3 to q27. It could be divided into two distinct portions, namely the critical region 1, which mainly overlaps the Xq21 band and the critical region 2, which spans from Xq23 to Xq27 [8, 9, 4]. POI was often associated either to interstitial deletions within Xq23–q27 or to balanced X;A translocations with breakpoints mapping to the Xq13–q21 region. However, most of breakpoints described in POI patients mapped to intragenic regions, consistently with models involving chromosome dynamic effects [10, 11], such as the “chromosomal position effect” (CPE) [12], possibly associated with X;A translocations [13]. Few characterizations of breakpoint regions in X;A balanced translocations presenting with POI led to the identification of interrupted genes mapping to the X chromosome [14]. One of these genes is *DIAPH2* (Diaphanous homolog 2 Drosophila), mapping to Xq21.33, which affects fertility in female flies by interfering with follicular cell division. This functional homology makes *DIAPH2* an excellent candidate for the POI, but its role in the etiology of the disorder is still unknown [15].

Another candidate for forms of familial and sporadic POI is *FMR1* (Fragile X Mental Retardation 1) gene, mapping to Xq27.3. It is known that amplification of the CGG triplet number above the normal range towards the premutation status of *FMR1* acts as a risk factor for DOR (Diminished ovarian reserve) [16] and POI/POF [17–19], with a ~ 20 % prevalence of POI in women with premutated alleles (especially those with 80-99 repeats) [20]. Furthermore, in a family with an interstitial duplication encompassing the *FMR1* gene, all females carrying duplicated *FMR1* underwent POF condition, suggesting that *FMR1* over-representation is associated to lower ovarian reserve as well as *FMR1* premutation.

Here we report molecular characterization and X-inactivation pattern in a girl with short stature, high levels of gonadotropins and POI, carrying a de novo balanced X;1 chromosome translocation. The analysis unravelled a mosaic partial inactivation of the translocated Xq chromosome trait, in addition to the fully skewed inactivation of the normal X chromosome. Two genes involved in ovarian deficiency, *DIAPH2* and *FMR1*, were found dysregulated in peripheral proband's lymphocytes.

Our results support the view that phenotypic anomalies of the proband might be caused by a mosaic functional alteration of some translocated genes, caused by the alteration in spatial nuclear organization, through a CPE mechanism, as a result of the chromosomal rearrangement.

## Case presentation

The proposita is a 10.5 year-old girl, second born of non-consanguineous healthy parents. Her elder brother is referred to be healthy and his stature is 150 cm at 14 years (5th centile for age) (National Center for Health Statistics in collaboration with the National Center for Chronic Disease Prevention and Health Promotion (2000) [21]), with pubertal development of PH3 G3 following Tanner stage. A third pregnancy hesitated in a spontaneous abortion. Her father is 169 cm whereas her mother is 150 cm high.

She was born at 39 gestational weeks, after normal pregnancy, by Caesarian section. Intrauterine growth retardation was detected during last weeks of gestation and her neonatal weight was 2.400 Kg, with a stature of 48 cm and an occipito-frontal circumference of 33 cm. In neonatal period, because of heart murmur, she underwent echocardiographic evaluation that evidenced an ostium secundum interatrial defect, spontaneously closed at 2 years. At 7 years slight hairs covered her back and arms, and overweigh was noted. Her parents reported slowdown in growth curve in the last 6 months despite a rapid increase in body weight.

Anthropometric values and clinical findings at 7 years were: stature 114.6 cm (3-10th centile), weight 25 kg (75th centile), BMI 19 (90-95th centile). Pubertal development was PH1 B1 following Tanner stage; genetic target was cm 153.5 + 6. At a further observation at 8 years, she presented with healthy condition, short stature (height cm 116 – below 3rd centile), overweight (weight kg 29 – 75th centile, BMI 21.55 – above 95th centile), hypertrichosis with slightly hairy legs and back, mild short neck, very mild valgus elbow, and small joint hyperlaxity, shortening of the fifth metacarpal with syndactyly of 2th and 3th of the toes.

At a first examination, laboratory tests evidenced: FSH: 12.3 mIU/ml; LH: 0.8 mIU/ml; Estradiol 10.2 pg/ml. Adrenal and thyroid profiles were within the normal range for age. Abdominal ultrasound revealed normal kidney, and pelvic echography showed reduced ovarian size range for age (right ovary 15 mm × 8 mm × 12 mm; left 13 mm × 7.4 mm × 10 mm). Because of hairiness increase, a new serum hormonal evaluation was performed at 7.5 years: laboratory tests showed elevated FSH values (31.3 mUI/ml) with normal prepuberal values of LH (0,7 mUI/ml) and Estradiol (13.2 pg/ml); a further pelvic ultrasound revealed infantile uterus and confirmed size reduced ovaries.

Analysis of *FMR1* triplet repeat sizes in the proband by repeat primed PCR [22] revealed sizes in the normal range (20 to 33 CGG repeats) (data not shown).

During 1-year follow-up period, a POI was suspected according to high FSH and LH levels and low Estradiol, inhibin B and AMH levels. Serum FSH and LH values increased to 179.90 mlU/ml and 40.30 mlU/ml respectively, while Estradiol decreased to 10 pg/ml. Inhibin B showed a level <20 pg/ml and AMH serum levels were 0.4 ng/ml, well below the normal range.

Bone age of 6 years was assessed by radiological examination of the left-hand wrist (Tanner e Whitehouse (TW2) method), despite a chronological age of 8 years. Stimulation tests for growth hormone (GH) reserve were performed because of deceleration of growth curve, increased gap from the stature target, and retardation of bone age. Testing for GH revealed a 1.4 ng/ml peak after arginine injection (0.5 g Arginina/kg in vein infusion in 30 minutes) and a 4.5 ng/ml peak after clonidine administration (100 mcg/m2 per os). Replacement therapy with rh-GH at standard dose for GHD patient was started (0.23 mg/kg/week). During GH-replacement therapy follow-up a gain of growth rate (25-50th centile) with achievement of target height in the first 6 months of therapy was observed. Any side effect was reported.

Pubertal induction with estroprogestin hormones was considered in long-term follow-up to prevent the ovarian failure that often occurs in majority cases before puberty and leads to infertility.

## Methods

### Chromosome analysis

High resolution karyotypes from peripheral blood and skin fibroblasts of the proband and her parents were performed according to standard methods.

Fluorescence in situ hybridization (FISH) analysis was performed using Whole Chromosome Paint (WCP) for chromosomes X and 1 and LSI *XIST* probe (MetaSystems, Altlussheim, Germany) on metaphase spreads and/or interphase nuclei according to manufacturer’s protocols. Multicolor banding (MCB) was performed using the multicolor banding DNA Probe Kit (MetaSystems, Altlussheim, Germany) according to manufacturer’s protocols [23]. A total of 100 metaphase spreads and/or 200 nuclei were analyzed.

Late-replicating chromatin was detected as previously described [24] using mouse monoclonal anti-BrdU (Invitrogen Molecular ProbesTM). WCP1 (MetaSystems, Altlussheim, Germany) was used to paint chromosome 1 in the same preparation. Fluorescence assay of 5-methylcytosine was performed according to the protocol described by Pfarr *et al.* [25]. Fluorescent images were analyzed using a fluorescence microscope (AxioImager.Z1 mot, Zeiss) with ISIS software imaging system (MetaSystems, Altlussheim, Germany) for image capturing and processing. Two hundred nuclei and metaphases were analyzed for each cell type.

Chromosomal position was evaluated by MCB and dual color WCP of chromosomes 1 and X. We simplified the 2D method described by Boyle *et al.* (2001) [26], identifying 2 regions in the nucleus, namely an internal region and an external one.

### Array-CGH analysis

Array-CGH of DNA from peripheral blood lymphocytes and skin fibroblasts of the proband and her parents was performed by CGX™-HD 4×180K (PerkinElmer Wallac, Turku, Finland) in accordance with manufacturer’s guidelines for the whole genome screening. The arrays were scanned using the InnoScan 710 and analyzed using Genoglyphix® software (Signature Genomics Spokane, WA), referring to the GRCh37/Hg19 Genome Assembly. Copy number variations were classified according to the Database of Genomic Variants [27], the DECIPHER Database [28], the UCSC Genome Browser [29] and the ISCA consortium [30].

### qRT-PCR

Total RNA from proband and control whole blood was extracted using TRIzol reagent (Gibco/BRL Life Technologies, Inc., Gaithersburg, MD) and was reverse transcribed using the iScript cDNA Synthesis kit (Bio-Rad Laboratories Inc., Hercules, CA, USA). Real-time PCR was performed using iQ Supermix SYBR Green 2× on a Bio-Rad iCycler according to the manufacturer’s protocols. PCR reactions were performed in triplicate. The primers (MWG Biotech, Ebersberg, Germany) used for amplification were:

- *DIAPH2*- For ACCCCAAACAACCCAACAT; Rev GGTGAAAATCGTTCCCTGTT;
- *FMR1*- For AGAAAAGTACCTGGGGTCACTG; Rev GCATCCTGATCCTCTCCATAAA.

Primer pairs were designed using the Primer 3 software [31] to obtain amplicons ranging from 100 to 150 base pairs. *GAPDH* housekeeping gene was chosen as reference gene. Expression values were normalized versus a pool of RNA from 3 healthy girls matching for age.

## Results

### Cytogenetic and molecular analysis

**Fig. 1:**
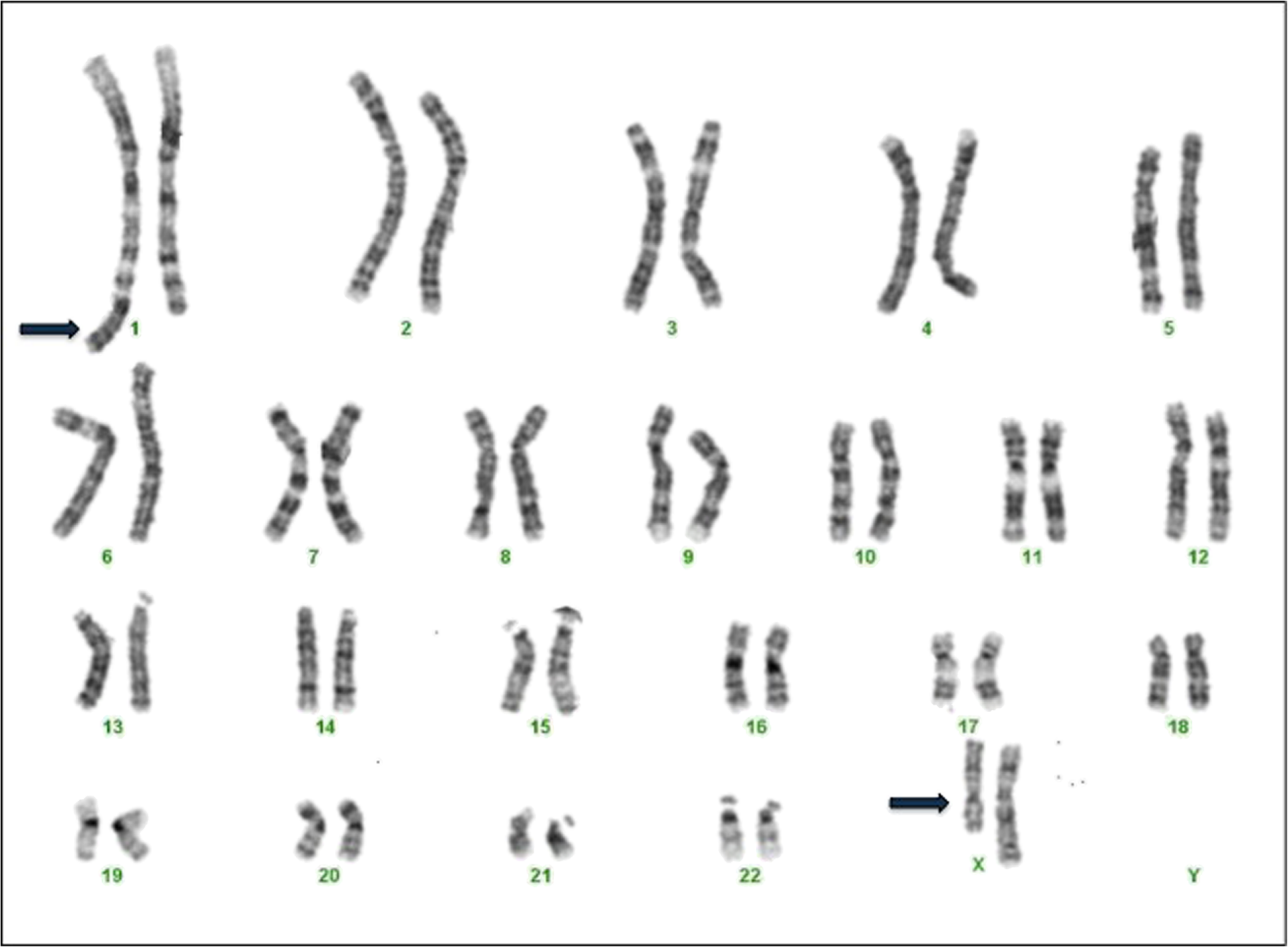
Patient G banded karyotype. Karyotype at 550 band resolution shows a reciprocal translocation between 1 and X chromosomes (blue arrows): t(X;1)(q21;q41)

Cytogenetic analysis of the proband, performed at 550 bands on G-banded metaphases from peripheral blood lymphocytes and skin fibroblasts, revealed a balanced translocation t(X;1)(q21;q41) (Fig. 1). FISH analysis using WCP probes for chromosomes X and 1 confirmed the conventional banding cytogenetic findings (data not shown).

**Fig. 2:**
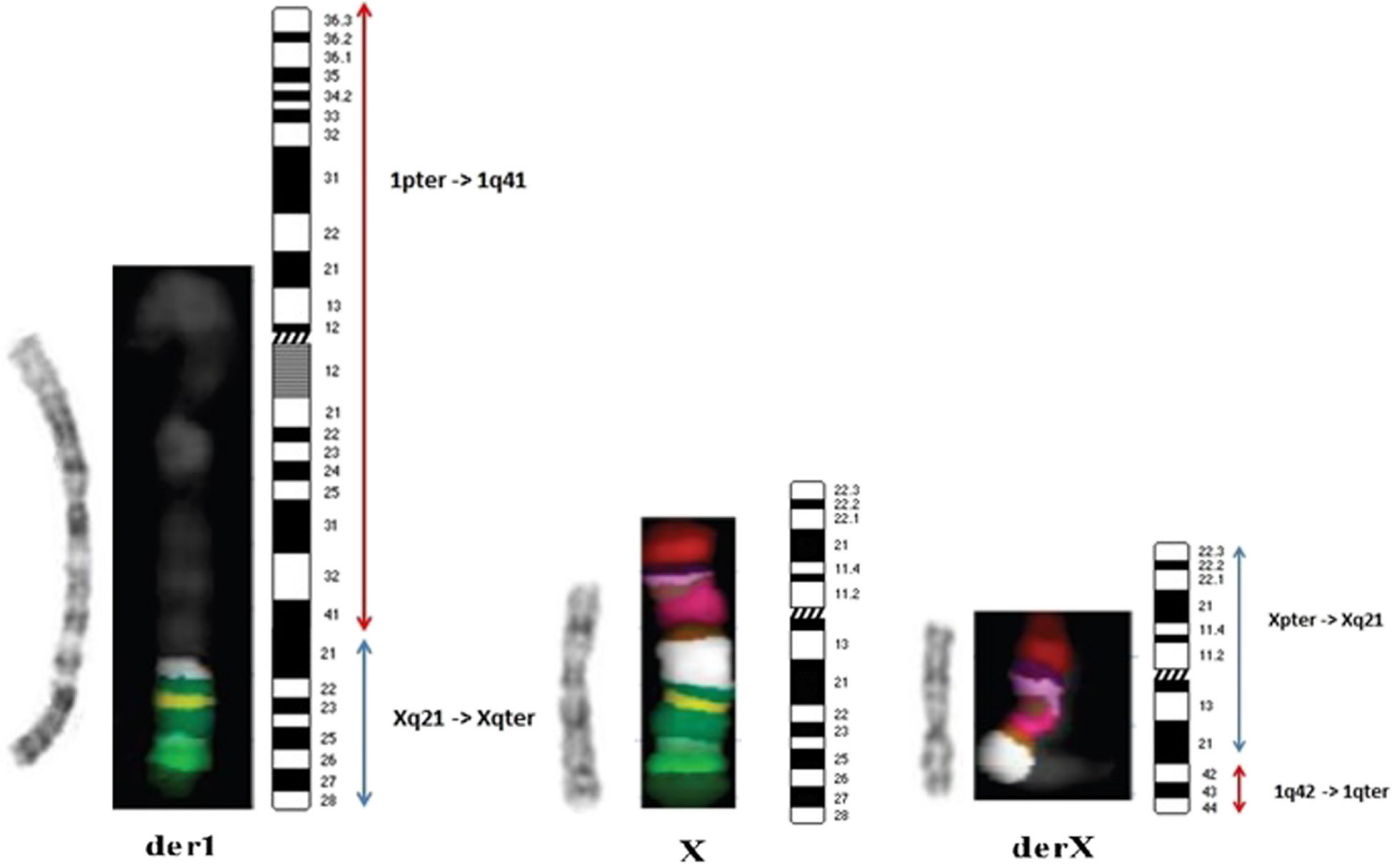
Multicolor Banding assay. G-Banding images, high resolution multicolor banding (MCB) and ideograms of the derivative 1 and derivative X chromosomes compared with the normal X chromosome. The region Xq21-qter translocated downstream the region 1q41 is shown

Multicolor banding (MCB) for chromosomes X and 1 was performed to further characterize the rearrangement and the breakpoints. It showed that the region Xq21-qter was translocated downstream the 1q41 band, giving rise to the derivative chromosome 1 (der(1)) and that the 1q42qter trait was translocated to the long arm of the X-chromosome, giving rise to the derivative X (der(X)) chromosome (Fig. 2).

**Fig. 3:**
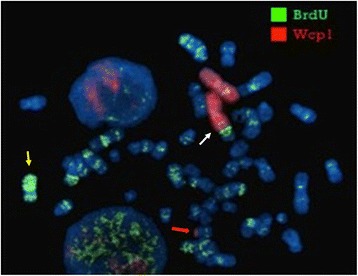
Late replication assay. Fluorescent BrdU assay, combined with WCP1 probe FISH, shows that the normal X chromosome was late replicating (yellow arrow). The late replication also interested the region Xq21-q22 translocated to der(1) (white arrow). Red arrow points to der(X)

Fluorescent BrdU assay, combined with WCP1 probe FISH, in both lymphocytes and fibroblasts, showed that the normal X chromosome was always late replicating and therefore inactivated. Interestingly, we also found that, in addition to the skewed 100 % inactivation of the normal X chromosome, part of the Xq21q22 trait, translocated to chromosome 1q41, was late replicating and therefore possibly inactivated in 30 % metaphases both in lymphocytes and skin fibroblasts (Fig. 3).

**Fig. 4:**
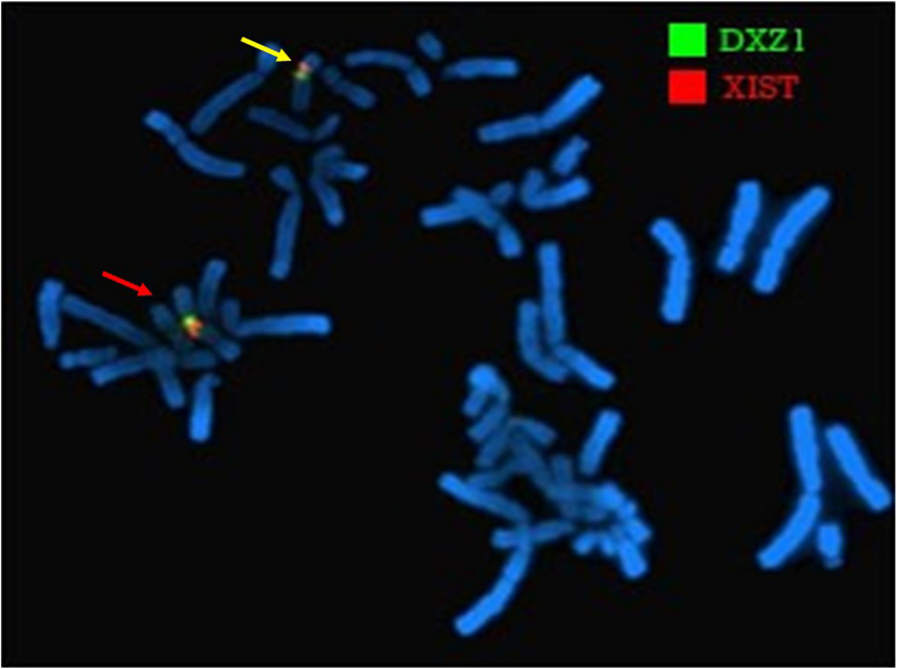
FISH analysis of *XIST*. FISH analysis using *XIST* probe shows the localization of the *XIST* gene on normal X chromosome (red arrow) and on the derivative X chromosome (yellow arrow)

FISH analysis using *XIST* probe (mapping to Xq13.2) showed signals on both normal X chromosome and der(X), thus excluding that the Xq inactivation observed on der(1) is *XIST*-mediated (Fig. 4).

To confirm this hypothesis we performed fluorescent 5-methylcytosine (5-Mc) assay that discriminates the state of heterocromatization in which the chromatin is densely methylated.

**Fig. 5:**
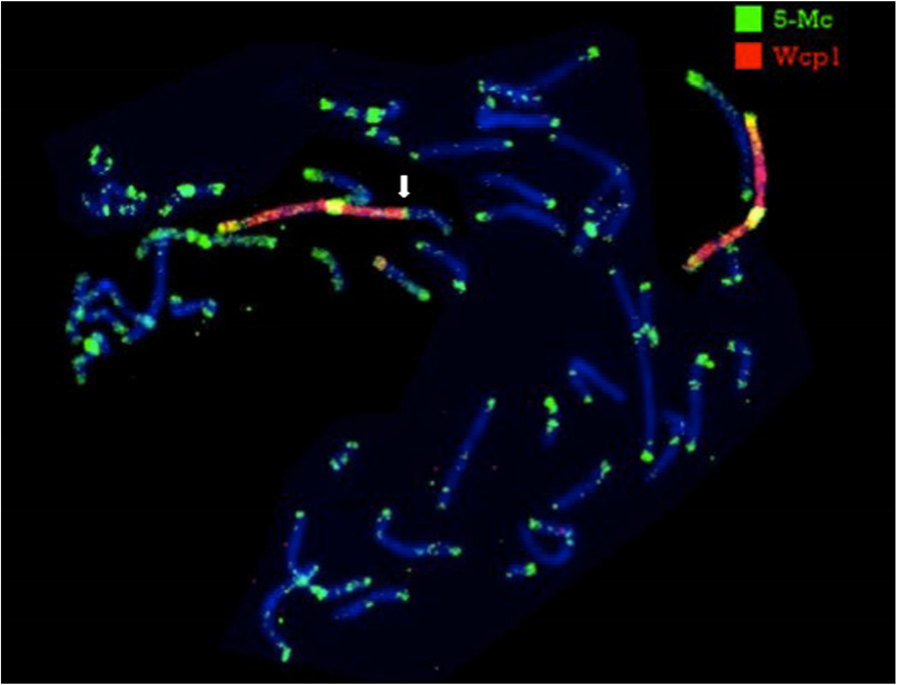
Fluorescent 5-methylcytosine (5-Mc) assay. 5-Mc antibody, combined with WCP1 probe FISH, shows that the X region involved in translocation was heterocromatized (white arrow)

This assay, combined with WCP 1 probe FISH, showed a hypermethylation, typical of constitutional heterochromatin, of the Xq21-q22 region of the der(1) in 30 % metaphases (Fig. 5). This situation might lead to functional mosaic aneusomy of some translocated genes and/or gene regulators in this region [32, 33].

**Fig. 6:**
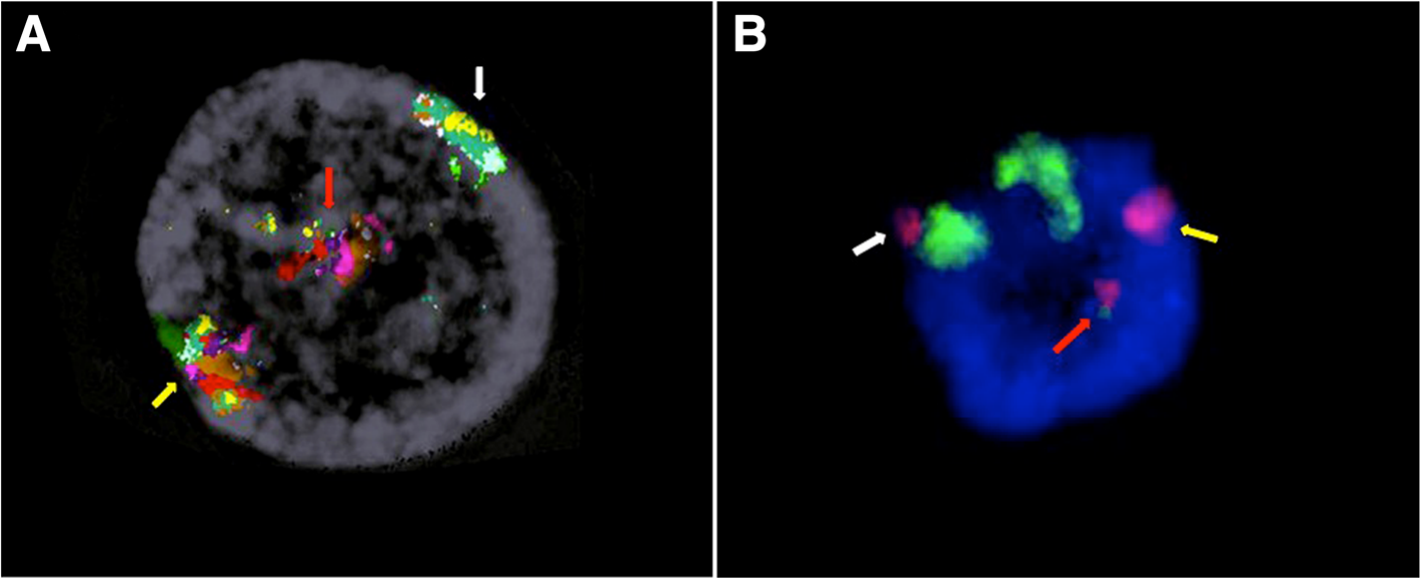
Nuclear positioning assay. 6A. Interphase Multicolor banding shows the normal X chromosome (yellow arrow) and the Xq21-q22 region on der(1) (white arrow) located peripherally in the observed nucleus, whereas der(X) is located centrally in the nucleus (blue arrow). 6B. Dual FISH using whole chromosome painting probes for chromosome 1 (green) and for chromosome X (red) shows the normal X chromosome (yellow arrow) and the Xq21-q22 region on der(1) (white arrow) located peripherally in the nucleus, whereas der(X) is located centrally in the nucleus

We next examined the nuclear positioning of the normal X and of the late replicating Xq21-q22 trait on der(1), performing interphase multicolor banding and dual DNA-FISH using whole chromosome paint probes of chromosomes X and 1. Both in skin fibroblasts and in lymphocytes, normal X was found peripherally in all analyzed nuclei, whereas the translocated Xq21-q22 region was located peripherally in 30 % of the observed nuclei (Fig. 6), suggesting that gene expression variation at the translocated region might be associated to its nuclear compartmentalization.

### Array-CGH analysis

The Oligo-array-CGH analysis of the proband unraveled only a likely benign partial monosomy of a 159Kb trait on 10q11.22, representing a copy number variant of uncertain clinical significance [27, 28, 30]. Array-CGH analysis excluded any other gain or loss of the proband’s genome (data not shown).

The complete chromosomal characterization, according to ISCN 2013, was as follows: 46,X,t(X;1)(Xpter- > Xq21::1q42- > 1qter;1pter- > 1q41::Xq21- > Xqter)dn.arr[hg19]10q11.22 (46,979,266-47,138,326)x1.

### qRT-PCR

**Fig. 7:**
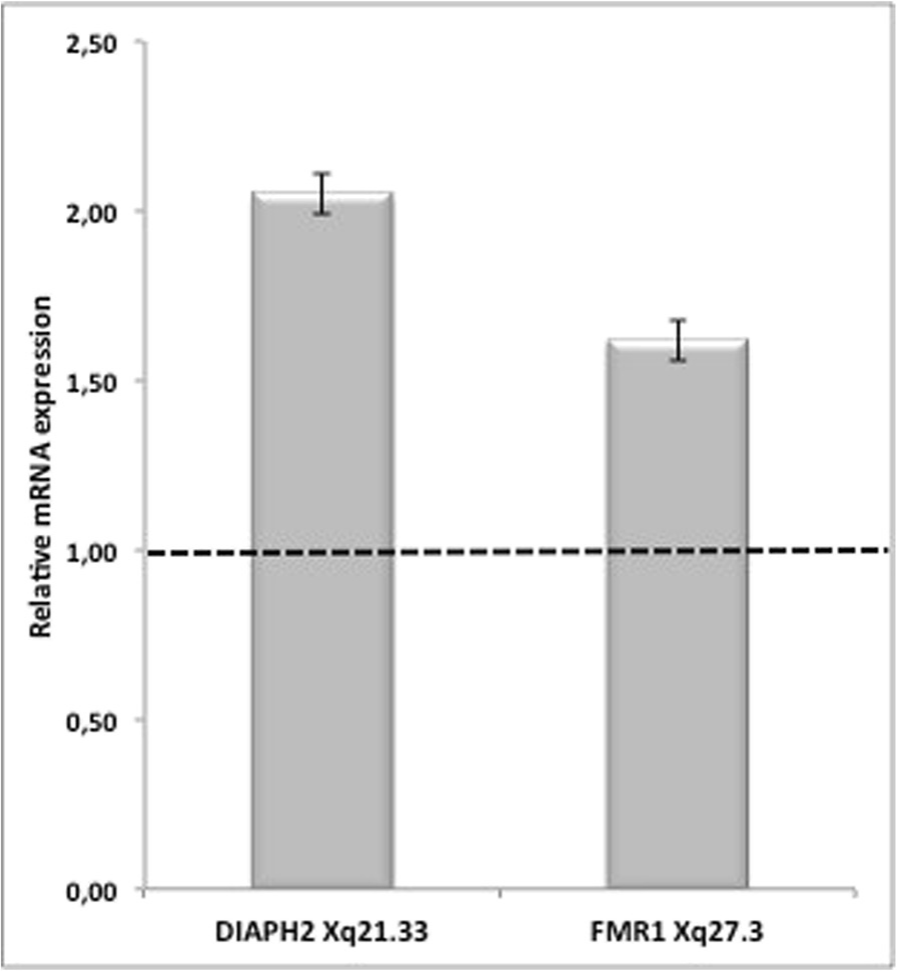
Gene expression analysis. qRT-PCR in proband versus control blood shows upregulation of *DIAPH2* gene (2,05) and of *FMR1* gene (1,62). Values represent the average determination ± SEM for 2 experiments carried out in triplicate. A pool of RNA from 3 healthy girls, matching for age was used as calibrator

Gene expression analysis by qRT-PCR on the RNA from lymphocytes of the proband showed upregulation of the *DIAPH2* gene, mapping to Xq21.33 (fold change = 2,05), and of the *FMR1* gene, mapping to Xq27.3 (fold change = 1,62), if compared to 3 age matching controls (Fig. 7).

## Discussion

Herein we describe a patient with high FSH and LH levels, pathologic GH stimulation arginine and clonidine tests, short stature, pterygium, ovarian dysgenesis, hirsutism and POI. By cytogenetic and molecular investigations we found a balanced X;1 translocation, with Xq21-q22 region translocated to 1q41 band. This region was found to be partially inactivated in 30 % metaphases both in fibroblasts and in lymphocytes, in addition to the skewed 100 % inactivation of the normal X chromosome. We also found that two genes involved in ovarian deficiency, *DIAPH2* and *FMR1*, were ovexpressed if compared with normal controls.

Some possible mechanisms to explain proband ovarian defect such as microdeletions close to the breakpoints or *XIST*-mediated inactivation of the autosomal region translocated to the der(X) chromosome have been excluded by array-CGH analysis and FISH analysis using *XIST* probe, which also excluded a *XIST*-mediated inactivation of der(1) segments. MCB analysis also excluded the involvement of other POI candidate genes such as *FGF16* gene and its syntenic region [7], which are not included in the translocated trait.

In agreement with other authors [15, 7] we propose CPE as alternative possible mechanism to justify the proband altered phenotype and in general POI phenotypes in cases of balanced X;autosome translocations without any evidence of gene dysruption. CPE can be caused by alterations in local chromatin structure due to the alterations in nuclear organization that may affect gene expression [34, 35] leading to variegated pattern of gene regulation [12, 13].

The study of X-chromosome inactivation in this case, using fluorescent BrdU assay and fluorescent 5-methylcytosine assay combined with WCP probe FISH supported the hypothesis that the alteration in spatial organization of the derivative chromosomes within the nucleus might be the cause of the dysregulation of expression of some X translocated genes, including *DIAPH2* and *FMR1*. The repositioning of the derivative chromosome within the nucleus to a more peripheral location, as identified by interphase MCB analysis and dual color whole chromosome painting, suggests that the variegated inactivation of the X translocated trait on der(1) could be associated with its nuclear compartmentalization with derived gene dysregulation, even though we cannot exclude that gene expression changes are caused by other factors of genome structure and function, such as replication timing, which also correlate with nuclear position and repositioning [36].

*FMR1* gene maps at Xq27 and is responsible for Fragile X syndrome, a form of X-linked mental retardation. The female premutation status of FMR1 gene acts as a risk factor for POI, POF and DOR pathogenesis [16]. A high variability of *FMR1* expression levels has been demonstrated in women carrying an *FMR1* premutation, even though the variance did not directly correlate to CGGn [37]. Our analysis demonstrated a 60 % overexpression of *FMR1* gene (Fig. 7) in our patient, in which molecular studies, previously performed, excluded the presence of *FMR1* premutation status.

As cellular toxicity and increased apoptosis have been demonstrated in case of *FMR1* over-expression, we cannot exclude that that POI phenotype could be partially caused by the high expression level of *FMR1*.

The other gene we found overexpressed in the proband, the *DIAPH2* gene [14], affects fertility in female flies, and has been found disrupted in a family with POI, though no mutation demonstrated its role in ovarian function to date. To our knowledge *DIAPH2* gene expression has never been investigated in other families with POI. This represents the first case in which POI is associated with an overexpression of *DIAPH2*. The patient here reported showed together with POI, other traits of a Turner like phenotype such as short stature, pterygium colli and hirsutism. A plausible explanation of all these clinical features in the proband should be a functional mosaic double inactivation of genes mapping to the Xq21-q22 region, as demonstrated by immunofluorescence assays, causing a functional Xq partial monosomy.

## Conclusions

Our results suggest that POI phenotype of the proband is caused by a dysregulation of the expression of some X-linked genes translocated to the chromosome 1, through either a mechanism of position effect variegation or an alteration in spatial nuclear organization, or both, as a result of chromosomal rearrangement. Specifically, perturbated genes within region Xq21-qter on der(1), such as *DIAPH2* and *FMR1*, may be causative candidate for gonadal dysgenesis and POI in our patient.

Further investigations will be aimed at both assessing the state of methylation of genes and promoters mapping to Xq21-q22 region, and searching for transcriptional regulators affecting the expression of those gene.

This is the second case in which we demonstrate an inactivation of X traits translocated to autosomes [13], possibly due to position effect. We suggest that such an inactivation might affect POI development as well as the position effect on autosomal genes proposed by other authors [15].

Epigenetic studies of patients with POI phenotype might be of considerable relevance to further understand the etiological mechanisms of POI.

## Consent

Written informed consent was obtained from the patient’s parents for publication of this Case report and any accompanying images. A copy of the written consent is available for review by the Editor-in-chief of this journal.

## Abbreviations

POI: Primary ovarian insufficiency
FSH: Follicle-stimulating hormone
LH: luteinizing hormone
POF: Premature ovarian failure
AMH: Anti-Mullerian hormone
CPE: Chromosomal position effect
DIAPH2: Diaphanous homolog 2 Drosophila
FMR1: Fragile X Mental Retardation 1
DOR: Diminished ovarian reserve
FISH: Fluorescence in situ hybridization
WCP: Whole Chromosome Paint
MCB: Multicolor banding
5-Mc: 5-methylcytosine
